# A Chemical Epigenetic Probe to Capture the Site-Specific DNA-Binding Protein Complex

**DOI:** 10.21203/rs.3.rs-5915426/v1

**Published:** 2025-03-11

**Authors:** Jiajun Zhu, Zhucui Li, Dongxiang Xue, Zihe Meng, Sida Shao, Julian Pulecio, Guoan Zhang, Danwei Huangfu, Todd Evans, Peter G. Schultz, Shuibing Chen

**Affiliations:** 1 Department of Surgery, Weill Cornell Medicine, 1300 York Ave, New York, NY, 10065, USA.; 2 Center for Genomic Health, Weill Cornell Medicine, 1300 York Ave, New York, NY, 10065, USA.; 3 Proteomics and Metabolomics Core Facility, Weill Cornell Medicine, New York, NY 10065, USA.; 4 Department of Chemistry, Scripps Research, San Diego, CA, USA.; 5 Developmental Biology Program, Sloan Kettering Institute, New York, NY, USA.

## Abstract

Site-specific DNA binding by proteins is critical for regulating genetic activity and cell fate decision. However, identifying proteins bound to specific genomic regions (e.g., promoter or enhancer) remains challenging. To address this, we developed a chemical epigenetic tool, named Site-specific non-canonical amino acid resolved Protein EnRichment (SUPER) system, incorporating a photo-crosslinking amino acid into nuclease-deficient dCas9 mutant. Human pluripotent stem cells (hPSCs) carrying SUPER enables the capture of proteins bound to, in theory, any genomic location, facilitating the study of the cell context-dependent DNA-protein interactions. Using SUPER, we identified OCT4/SOX2/CARHSP1 complex binding to the *NANOG* promoter to maintain pluripotency in hPSCs. During ectoderm differentiation, ZIC2 acts as a competitive inhibitor, binding the same promoter to downregulate *NANOG* expression and promote differentiation. Additionally, SUPER identified ZNF8 binding to the distal regulatory region of *OCT4* and maintain naïve pluripotency. In summary, SUPER provides a robust system for uncovering the cell context-dependent, site-specific genome regulators, offering valuable insights into gene regulation networks driving cell fate transitions.

The site-specific binding of proteins to the genome regulates gene transcription and controls cell fate decision^1^. Both computational and experimental approaches such as chromatin immunoprecipitation sequencing (ChIP-seq) have been developed to identify DNA regions bound by specific transcription factors (TFs) or other regulatory proteins. However, few tools exist to identify protein complexes bound to specific sequences or regions in the genome. Nuclease-deficient Cas9 (dCas9) protein and a biotinylated version have been used to purify specific genomic regions^2^ and identify the chromatin interactions with cis-elements and super-enhancers^3^, respectively. These methods, however, rely largely on non-covalent interactions, which are typically weak, transient, and susceptible to disruption.

The ability to genetically encode non-canonical amino acids (ncAAs) in organisms allows one to site-specifically modify proteins with biological probes in a way not possible using the canonical twenty amino acids^4^. This approach uses engineered orthogonal tRNA/tRNA synthetase pairs that recognize a stop codon (e.g., amber or frameshift codon) and incorporate a ncAA at a specific site in the protein sequence. The Pyrrolysyl-tRNA synthetase (PylRS)/tRNA^Pyl^ pairs from archaea species *Methanosarcina barkeri* (*Mb*) and *Methanosarcina mazei* (*Mm*) are among the widely used since they are orthogonal in both *E. coli* and mammalian cells^5,6^. To date, over three hundred distinct ncAAs have been incorporated into proteins and have been used to study protein function, protein-protein interactions, live-cell imaging, and cellular signaling. Several photo-crosslinking ncAAs have been incorporated into proteins^7,8^ and nonselectively form covalent bonds with nearby interactors upon irradiation, providing sensitive tools to map dynamic biomolecular interactions *in vitro and in vivo*.

To probe cell-context dependent, site-specific DNA-protein binding, we developed human pluripotent stem cells (hPSCs), carrying dCas9 with an incorporated photo-crosslinking ncAA, (S)-2-amino-6-((2-(3-methyl-3H-diazirin-3-yl)ethoxy)carbonylamino)hexanoic acid (AbK). This ncAA is structurally similar to lysine, but contains a diazirine group in its side chain^9^, which allows efficient probing of protein-protein interactions with minimal structural perturbation. With the appropriate small guide RNA (sgRNA), dCas9 can, in theory, carry the photo crosslinking AbK to most regions within the human genome. Finally, the host hPSCs, including human embryonic stem cells (hESCs) and induced pluripotent stem cells (iPSCs) have the potential to differentiate into various cell types, enabling the study of cell context dependent site-specific DNA-protein interactions.

## Results

### Development of Site-specific ncAA-resolved Protein EnRichment (SUPER) System.

**Extended Data Fig. 1.**
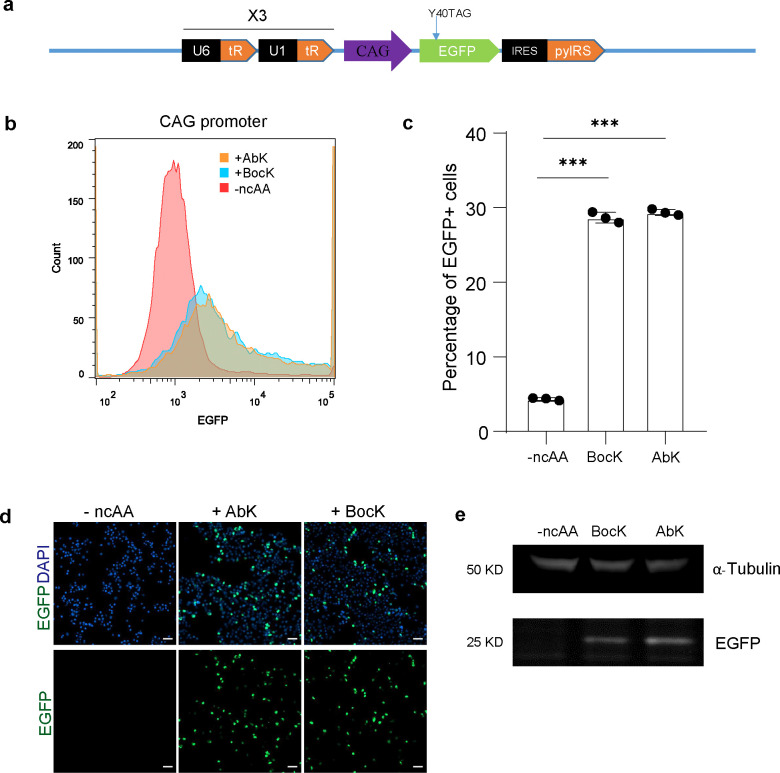
Development of SUPER system. (**a**) Schematic of the construct to incorporate ncAA in EGFP. (**b** and **c**) Flow Cytometry analysis (b) and quantification (c) of EGFP expression in hESCs carrying EGFP^Y40TAG^ cultured in control (−ncAA), AbK and Bock conditions. (**d** and **e**) Immunostaining (d) and (e) western blot analysis of EGFP expression in hESCs carrying EGFP^Y40TAG^ cultured in control (−ncAA), AbK and Bock conditions. Data represent the mean ± s.d. *P* value was calculated by Student’s t-test, ***P<0.001.

To incorporate Abk into proteins in hPSCs, H9 hESCs were first transfected with a construct containing mutant the pylRS/tRNA pair (EGFP^Y40TAG^ H9 hESCs, Extended Data Fig. 1a) along with a Y40TAG EGFP reporter^8^. In this system, robust EGFP expression is observed only upon successful incorporation of the ncAA. After transfection, EGFP^Y40TAG^ H9 hESCs were treated with AbK and BocK, a non-reactive lysine analogue control. Robust EGFP (Y40TAG) expression was observed in the presence of either AbK or BocK. Flow cytometry revealed that 37.9% and 39.2% of EGFP^Y40TAG^ H9 hESCs expressing EGFP in the presence of AbK and Bock, respectively (Extended Data Fig. 1b, c). Both immunostaining (Extended Data Fig. 1d) and western blotting analysis (Extended Data Fig. 1e) further validated the efficient incorporation of AbK and BocK in EGFP in EGFP^Y40TAG^ H9 hESCs.

**Extended Data Fig. 2.**
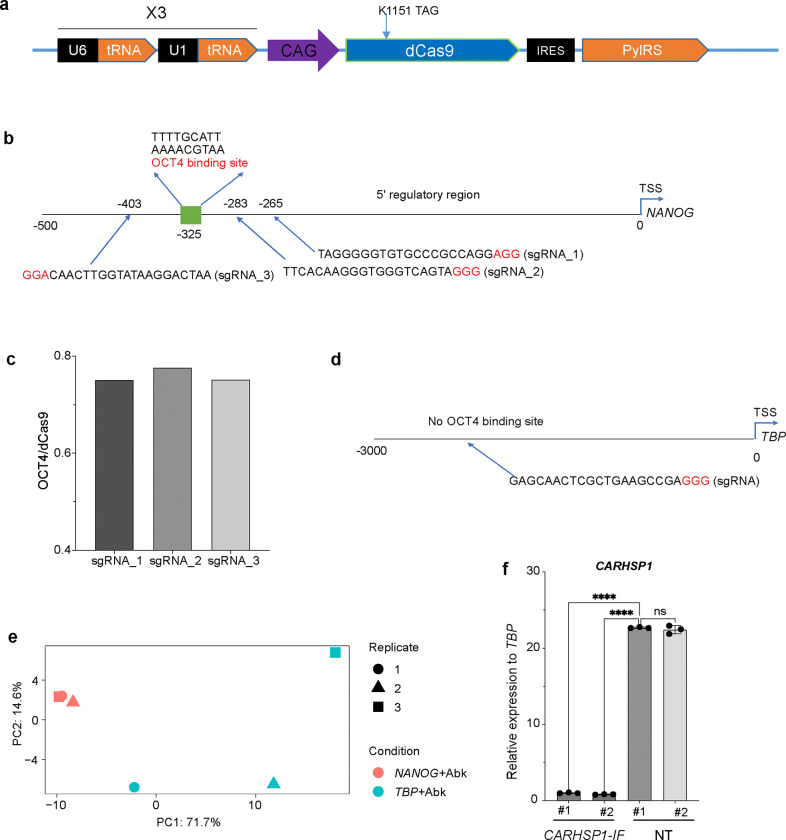
SUPER captures proteins binding to the *NANOG* promoter at hESC stage. (**a**) Schematic of SUPER construct. (**b**) Schematic of *NANOG* promoter region and sgRNAs. (**c**) Relative amount of OCT4 protein captured at *NANOG*+AbK condition using three different sgRNAs. (**d**) Schematic of the *TBP* promoter region and sgRNAs. (**e**) PCA plot of proteins captured by *NANOG*+AbK and *TBP*+AbK. (**f**) qRT-PCR analysis of *CARHSP1* expression in *CARHSP1-IF* hESCs. Data represent the mean ± s.d. *P* value was calculated by one-way ANOVA, ****P<0.0001, ns, a non-significant difference.

**Fig. 1.**
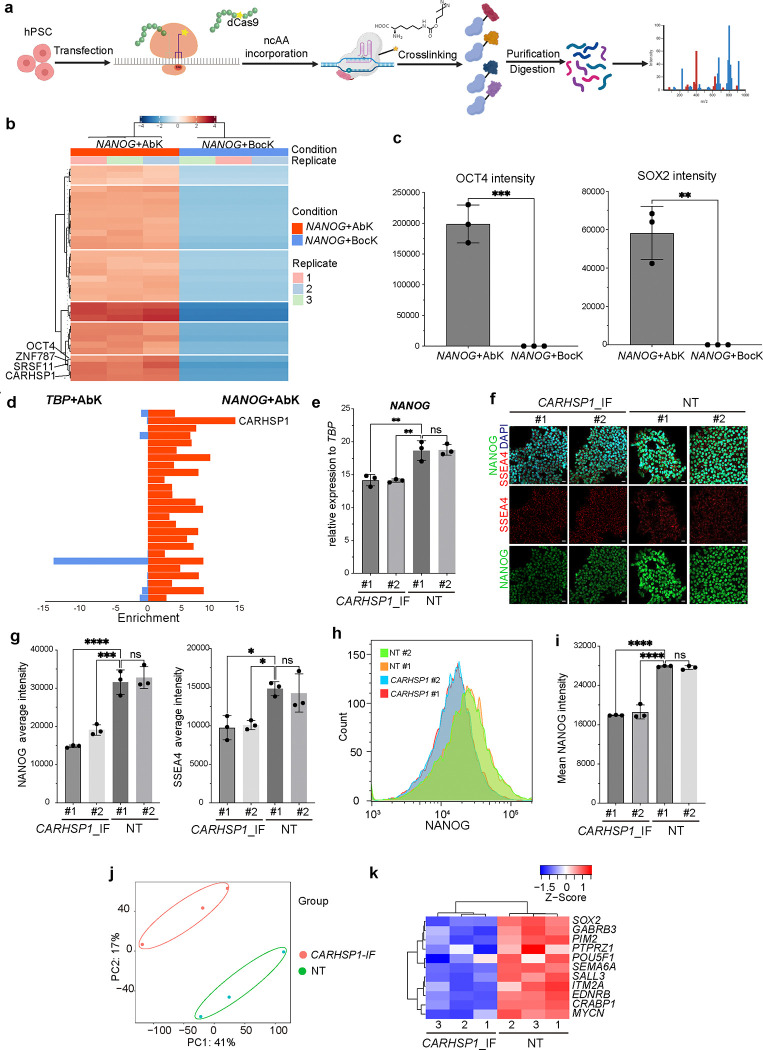
A chemical epigenetic tool captures the protein complex binding to the *NANOG* promoter at the hESC stage. (**a**) Scheme of the SUPER system. (**b**) Heatmap showing the proteins specifically captured by *NANOG*+Abk in H9 hESCs. (**c**) Bar plots displaying the abundance of OCT4 and SOX2 captured at *NANOG*+Abk and *NANOG*+Bock conditions. (**d**) Enrichment scores of top hits identified in (b) in *NANOG*+Abk and *TBP*+Abk conditions. (**e**) qRT-PCR analysis of *NANOG* expression in *CARHSP1-IF* and NT hESCs. (**f**, **g**) Immunostaining (f) and quantification (g) of NANOG and SSEA4 in *CARHSP1-IF* and NT hESCs. (**h, i**) Flow cytometry analysis (h) and quantification (i) of NANOG expression in *CARHSP1-IF* and NT hESCs. (**j**, **k**) PCA plot of whole transcriptomes (j) and heatmap of pluripotency genes (k) in *CARHSP1-IF* and NT hESCs. Data represent the mean ± s.d. *P* values of figures were calculated by one-way ANOVA, *P<0.05, **P<0.01, ***P<0.001, ****P<0.0001, ns, a non-significant difference.

Next, we developed the SUPER system using a dCas9 variant containing Abk to identify proteins that bind directly or indirectly to a specific genomic region (Fig. 1a). We first mutated Cas9 protein to a nuclease-deficient form by introducing two site mutations (D10A and H840A)^10^. Next, we mutated the K1151 residue of dCas9, to an amber stop codon (dCas9 K1151TAG). We chose K1151 site based on previous studies that compared various sites and demonstrated that the modification at K1151 does not impact Cas9 production ^11^. The dCas9 K1151TAG and the PylRS mutant recognizing AbK and Bock were cloned into a single construct to create SUPER system (Extended Data Fig. 2a). H9 hESCs were then transfected with SUPER system and sgRNAs, to generate H9^SUPER^ hESCs. Upon addition of AbK and exposure to long-wave UV light, an active carbene is formed from the diazirine, enabling covalent capture of proteins proximal to the targeted DNA region (Fig. 1a).

To validate the utility of the SUPER system, we designed three sgRNA targeting the promoter region of *NANOG*, which contains a known OCT4 binding site^12^. H9 hESCs were transfected with the SUPER system and the sgRNAs to create H9^SUPER_*NANOG*^ hESCs (Extended Data Fig. 2b, Table 1). After treating H9^SUPER_*NANOG*^ hESCs with ncAAs for two days, the cells were irradiated, and cell lysates were analyzed by mass spectrometry (MS). We first compared three sgRNAs targeting the *NANOG* promoter region, each within 100 bp of the known OCT4 binding site (Extended Data Fig. 2b) and assessed the proteins captured under AbK condition. Since OCT4 is known to bind to this *NANOG* promoter region^12^, we compared the amount of OCT4 captured using each sgRNA and chose sgRNA_2, located 42 bp from the target site, as it pulled down the highest amount of the OCT4 (Extended Data Fig. 2c).

Next, we compared the proteins captured using sgRNA_2 by AbK or non-photoactive BocK control. Most of proteins captured by BocK control were also detected at AbK condition, suggesting that the protein pulled down at Bock condition likely result from nonspecific binding. Therefore, we decided to focus on the proteins that were specifically captured at AbK condition (Fig. 1b). Among the identified proteins, OCT4 (Fig. 1c), previously shown to bind to *NANOG* promoter region^12^, was prominently detected. Additionally, we identified another pluripotency marker, SOX2 (Fig. 1c), which was detected in both groups but significantly enriched in the AbK group, suggesting that OCT4 and SOX2 may form a protein complex to regulate *NANOG* expression, consistent with previous studies^12^. To test the specificity of the SUPER system, we also designed a sgRNA targeting the promoter region of the housekeeping gene *TBP* as a control (Extended Data Fig. 2d, Table 1). We observed distinct clustering of proteins captured using *NANOG+*AbK and proteins captured using *TBP*+Abk (Extended Data Fig. 2e).

We next focused on the proteins that are highly enriched in *NANOG+*AbK, but not *TBP*+Abk conditions, the most abundant one is CARHSP1, a member of the cold-shock domain protein family that functions as a transcriptional or translational regulator (Fig. 1d)^13^. To investigate the biological roles of CARHSP1, we used an inducible H1 dCas9-KRAB system to suppress *CARHSP1* (*CARHSP1-IF*) expression in H1 hESCs (Table S1). qRT-PCR confirmed efficient silencing of *CARHSP1* expression in *CARHSP1-IF* hESCs, respectively (Extended Data Fig. 2f). Silencing of *CARHSP1* expression downregulated *NANOG* mRNA expression (Fig. 1e). NANOG protein expression also decreased in *CARHSP1-IF* hESCs as indicated by both immunocytochemistry (Fig. 1f, g) and flow cytometry (Fig. 1h, i). RNA-seq analysis showed that *CARHSP1-IF* hESCs cluster separately from nontargeting control (NT) hESCs (Fig. 1j), with the downregulation of many pluripotency genes (Fig. 1k). Finally, the pluripotency marker, SSEA-4, is also downregulated in *CARHSP1-IF* hESCs (Fig. 1f, g). Taken together these results suggest suppression of *CARHSP1* impairs pluripotency through downregulating *NANOG* transcription.

### SUPER identifies the cell context-dependent binding of ZIC2 to *NANOG* promoter.

**Extended Data Fig. 3.**
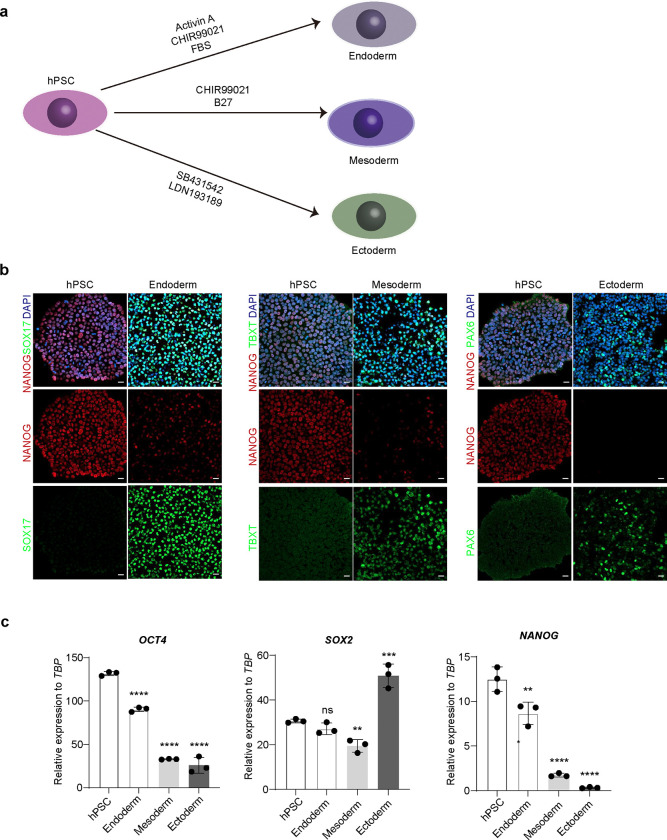
Characterization of three germ layer differentiation from hPSCs. (**a**) Schematic of three germ layer differentiation. (**b**) Immunostaining of pluripotency and lineage-specific marker expression in hPSC-derived endoderm, mesoderm, and ectoderm. (**c**) qRT-PCR analysis of *OCT4*, *SOX2* and *NANOG* expression in hPSC-derived endoderm, mesoderm, and ectoderm. Data represent the mean ± s.d. *P* value was calculated by one-way ANOVA, **P<0.01, ***P<0.001, ****P<0.0001, ns, a non-significant difference.

**Extended Data Fig. 4.**
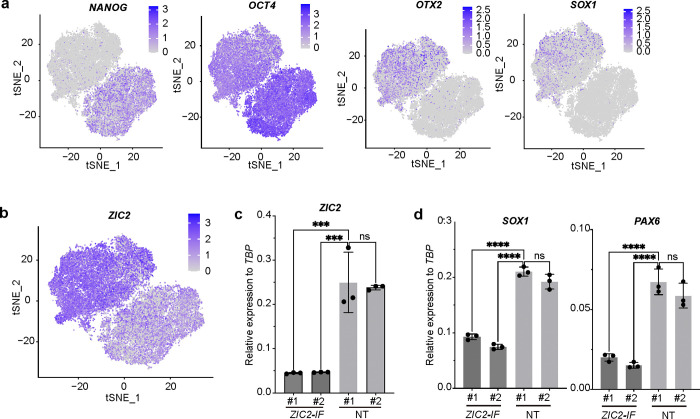
SUPER identifies ZIC2 as a competitive repressor binding to the *NANOG* promoter and controlling ectoderm differentiation. (**a**) tSNE plots of pluripotency genes (*NANOG, OCT4*) and ectoderm genes (*OTX2, SOX1)* of Perturb-seq analysis. (**b**) tSNE plots of *ZIC2* of Perturb-seq analysis. (**c**) qRT-PCR analysis of *ZIC2* expression in *ZIC2-IF* and NT hESCs. (**d**) qRT-PCR of expression of *SOX1* and *PAX6* expression in *ZIC2-IF* and NT hESCs upon ectoderm differentiation condition. Data represent the mean ± s.d. *P* value was calculated by one-way ANOVA, ***P<0.001, ****P<0.0001.

**Figure 2.**
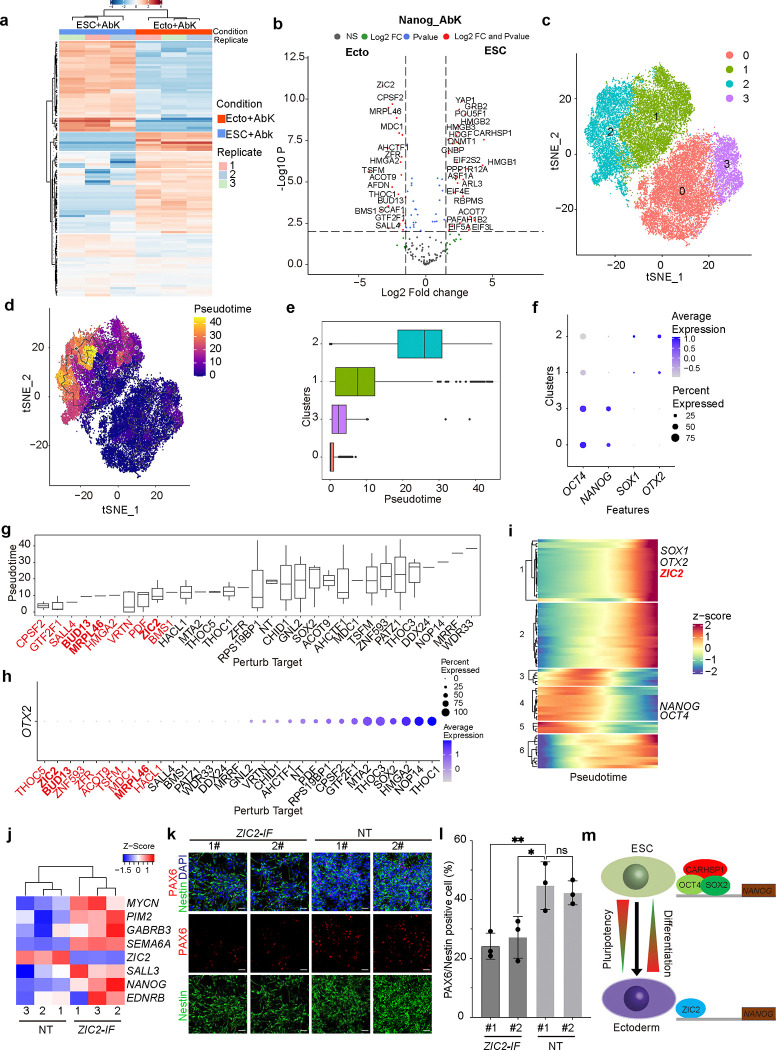
SUPER identifies ZIC2 as a competitive repressor binding to the *NANOG* promoter and controlling ectoderm differentiation. (**a**) Heatmap of relative abundance of proteins binding to *NANOG* promoter at hESC (ESC+AbK) and ectoderm (Ecto+AbK) stages, captured by the SUPER system. (**b**) Volcano plot showing proteins captured by *NANOG*+Abk at hESC versus ectodermal stages. (**c**) tSNE plot of Perturb-seq results. (**d**) Trajectory analysis of pseudotime ordering. (**e**) Box plot of pseudotime distribution of each cluster. (**f**) Dot plot of pluripotent markers, including *OCT4* and *NANOG*, and ectoderm markers, including *SOX1* and *OTX2*, in each cluster. (**g**) Box plot showing cells carrying sgRNAs against perturbation targets along pseudotime. (**h**) Dot plot of *OTX2* expression in cells carrying sgRNAs against perturbation targets. (**i**) Heatmap of perturbed gene expression along pseudotime. (**j**) qRT-PCR analysis of *NANOG* expression in *ZIC2-IF* and NT hESCs. (**k, l**) Immunostaining (k) and quantification (l) of the *SOX1* and *PAX6* expression in *ZIC2-IF* and NT hESCs. (**m**) Scheme of the proposed mechanism. Data represent the mean ± s.d. *P* values of figures were calculated by ANOVA, *P<0.05, **P<0.01, ***P<0.001, ****P<0.0001, ns, a non-significant difference.

We next used the SUPER system to study cell context-dependent binding at the *NANOG* promoter. As a key pluripotency marker, *NANOG* expression decreases during differentiation into endoderm, mesoderm, and ectoderm lineages, with the lowest expression observed in ectodermal cells (Extended Data Fig. 3a, b, c). Therefore, we compared protein binding at the *NANOG* promoter in both hESCs and hESC-derived ectodermal cells (Fig. 2a). We focused on the top differentially enriched proteins between hESCs and ectoderm stages (Fig. 2b) and performed Perturb-seq to validate candidate proteins. In brief, we designed four sgRNAs targeting each gene (Table S2) and generated lentiviral constructs carrying the CRISPR/Cas9 and sgRNA library. After infection and sorting, hESCs carrying the lentiviral library and their ectoderm derivatives were analyzed using scRNA-seq. tSNE analysis identified four major cell clusters (Fig. 2c). Trajectory analysis (Fig. 2d, E) revealed the progression of hESCs to ectoderm differentiation along pseudotime, with a decrease in pluripotency markers, including *NANOG* and *OCT4*, and an increase in ectoderm markers, such as *OTX2* and *SOX1* (Fig. 2f and Extended Data Fig. 4a).

Next, we ranked the cells along pseudotime and found that cells carrying sgRNAs targeting *CPSF2*, *GTF2F1*, *SALL4*, *BUD13*, *MRPL46*, *HMGA2*, *VRTN, PDF*, *ZIC2* and *BMS1* (top 10) are enriched in the early pseudotime (Fig. 2g). We also ranked cells based on expression of the ectoderm marker, *OTX2*. The top 10 perturbed genes enriched in cells with low OTX2 expression included *THOC5, ZIC2, BUD13, ZNF593, ZFR, ACOT9, TSFM, MDC1, MRPL46,* and *HACL1* (Fig. 2h). Three target genes, *ZIC2*, *BUD13*, and *MRPL46*, were enriched using both ranking methods. We then examined the expression patterns of these perturbed genes along pseudotime; only *ZIC2* exhibited increased expression during ectoderm differentiation (Extended Data Fig. 4b), mirroring the trends of ectoderm marker genes, *SOX1* and *OTX2* (Fig. 2i).

To validate the impact of ZIC2 on pluripotency and ectoderm differentiation, we silenced the expression of ZIC2 (*ZIC-IF*) at hESC stage using an inducible H1 dCas9-KRAB line (Table S1). qRT-PCR confirmed the efficient suppression of *ZIC2* in *ZIC-IF* hESCs (Extended Data Fig. 4c). *ZIC-IF* hESCs maintain *NANOG* expression under ectoderm differentiation conditions (Fig. 2j) and silencing of *ZIC2* blocks ectoderm differentiation, as indicated by the decreased expression of the ectoderm marker genes, *SOX1* and *PAX6* (Fig. 2k, 2l, Extended Data Fig. 4d). Taken together, these experiments identified an OCT4/SOX2/CARHSP1 protein complex that binds the *NANOG* promoter at the hPSC stage to maintain pluripotency. As cells differentiate toward ectoderm, ZIC2 acts as a competitive inhibitor, binding to the *NANOG* promoter to downregulate NANOG expression and promote ectoderm differentiation (Fig. 2m).

### Using SUPER to identify protein binding to the OCT4-distal enhancer in naïve and primed hESCs.

**Extended Data Fig. 5.**
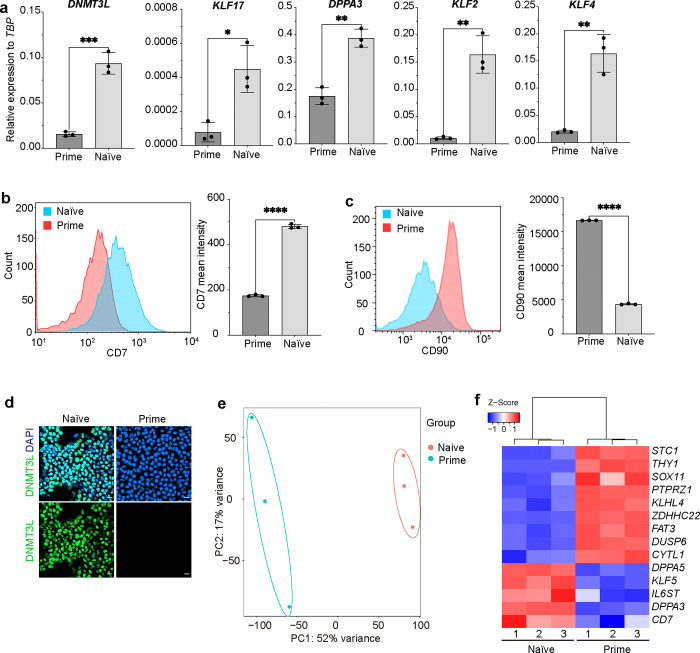
Characterization of naïve and primed hESCs. (**a**) qRT-PCR analysis of *DNMT3L*, *KLF17*, *DPPA3*, *KLF2* and *KLF4* expression in naïve and primed hESCs. (**b**) Flow Cytometry analysis of CD7 (B) and CD90 (**c**) expression and quantification in naïve and primed hESCs. (**d**) Immunostaining of DNMT3L expression in naïve and primed hESCs. (**e**) PCA analysis of whole transcriptome of naïve and primed hESCs. (**f**) Heatmap of marker gene expression in naïve and primed hESCs. Data represent the mean ± s.d. *P* value was calculated by one-way ANOVA, *P<0.05, **P<0.01, ***P<0.001, ****P<0.0001, ns, a non-significant difference.

In addition to promoter regions, we also used SUPER to identify proteins binding to enhancer regions. We focused on the distal enhancer region of OCT4, which is differentially regulated in primed and naïve hESCs^14^. Specifically, the proximal enhancer region drives OCT4 expression at the primed hESC stage, while the distal regulatory region is active in naïve hPSCs. H9^SUPER^ hESCs were transfected with sgRNAs targeting the distal enhancer locus of *OCT4* (Table 1), and photo-crosslinking was conducted in both primed and naïve hESCs. hESC cell status was confirmed by the expression of naïve or primed hESC markers, including *DNMT3L, KLF17, DPPA3, KLF2*, and *KLF4*, as determined by qRT-PCR (Extended Data Fig. 5a), CD7, as indicated by flow cytometry (Extended Data Fig. 5b, c), and DNMT3L as indicated by immunostaining (Extended Data Fig. 5d). RNA-seq further validated the primed and naïve pluripotency status (Extended Data Fig. 5e, f).

**Extended Data Fig.6.**
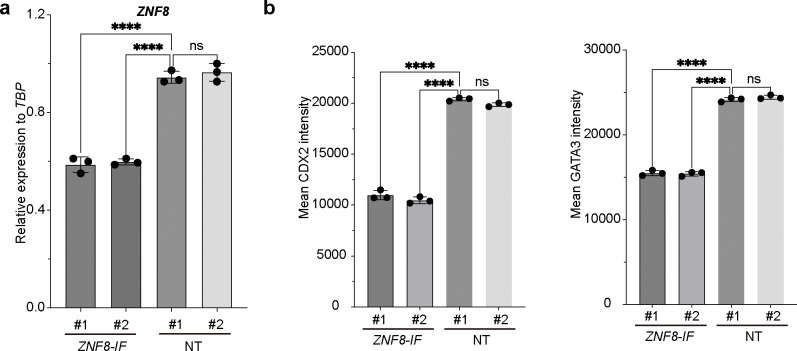
Characterization of *ZNF8-IF* naïve hESCs. (**a**) qRT-PCR analysis of *ZNF* expression in *ZNF-IF* and NT naïve hESCs. (**b**) Quantification of mean CDX2 and GATA3 expression in *ZNF8-IF* and NT naïve hESC-derived TSCs based on flow cytometry analysis. Data represent the mean ± s.d. *P* value was calculated by one-way ANOVA, ****P<0.0001.

**Figure 3.**
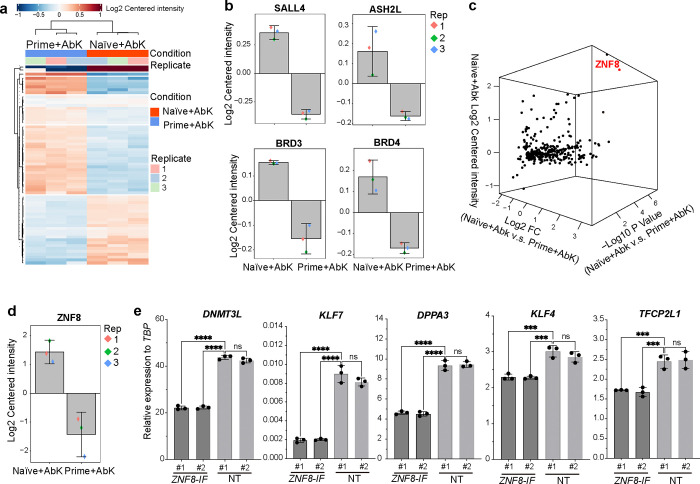
SUPER captures ZNF8 binding to the *OCT4* distal enhancer region. (**a**) Heatmap of proteins captured by SUPER at Naïve+AbK and Primed+Abk conditions. (**b**) Bar plots showing the relative abundance of SALL4, ASH2L, BRD3 and BRD4 in Naïve+AbK and Primed+Abk conditions. (**c**) 3D PCA plot of highly enriched proteins, with the X-axis representing Log2 fold change (FC) of Naïve+AbK versus Primed+AbK conditions, the Y-axis representing Log10 P-value of Naïve+AbK versus Primed+AbK conditions, and the Z-axis representing relative protein abundance in Naïve+AbK. (**d**) Bar plots displaying the relative abundance of ZNF8 in Naïve+AbK versus Primed+Abk conditions. (**e**) qRT-PCR of the expression of naïve pluripotency markers, including *DNMT3L, KLF7, DPPA3, KLF4* and *TFCP2L1* in *ZNF8 IF* and NT naïve hESCs. Data represent the mean ± s.d. *P* values of figures were calculated by one-way ANOVA, ***P<0.001, ****P<0.0001, ns, a non-significant difference.

MS analysis was used to identify proteins binding to the OCT4 distal enhancer region in primed versus naïve hPSCs (Fig. 3a). We observed enrichment of known OCT4 regulators, i.e., SALL4, in naïve ESCs but not primed ESCs (Fig. 3b), which is consistent with previous studies^15^. Additionally, several co-factors, including ASH2L, BRD3, and BRD4, which are known to drive enhancer activation and transcription, were also enriched under naïve conditions, indicating an active chromatin state at the distal enhancer region in naïve hESCs (Fig. 3b). Next, we focused on proteins that are enriched in the Naïve+AbK groups, but not Primed+AbK groups, and identified ZNF8 (Fig. 3c, d). An inducible H1 dCas9-KRAB line was used to suppress the gene encoding the candidate protein (Table S1), and qRT-PCR confirmed the successful silencing in *ZNF8* in *ZNF8-IF* hESCs (Extended Data Fig. 6a). The expression levels of several key naïve pluripotency markers, including *DNMT3L, KLF7, DPPA3, KLF4, TFCP2L4*, were reduced in *ZNF8-IF* naïve hESCs compared to naïve hESCs carrying non-targeting sgRNA (NT naïve hESCs) as indicated by qRT-PCR (Fig. 3e).

### ZNF8 functions as a key transcription factor controlling naïve pluripotency and blastoid formation.

**Figure 4.**
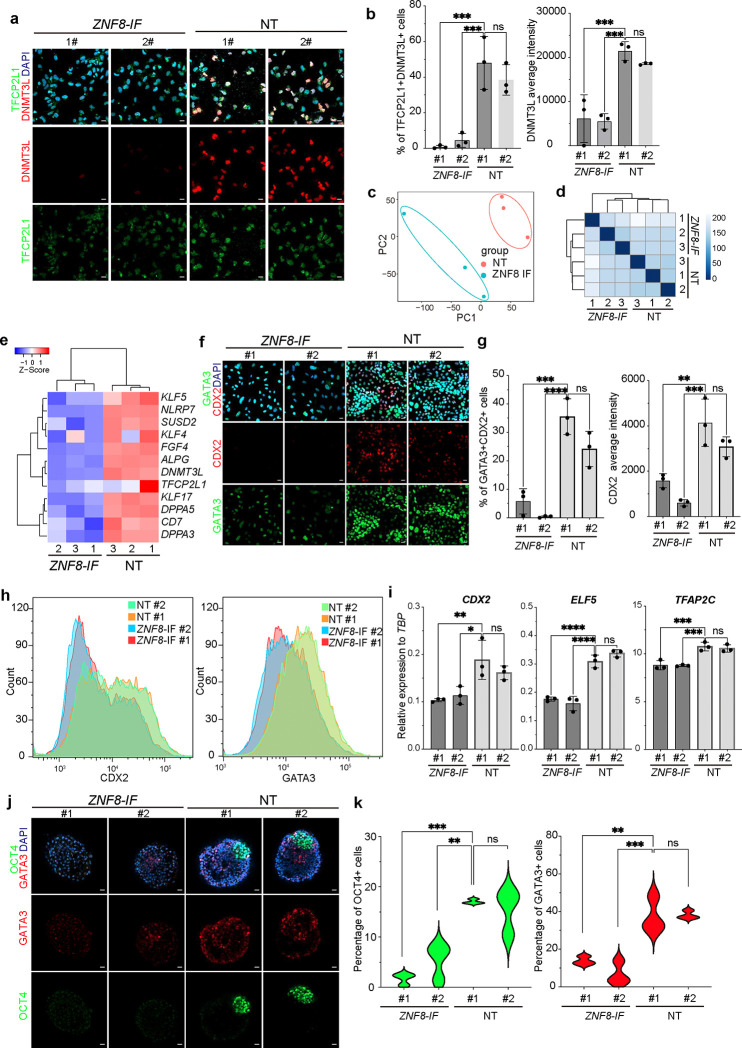
Silencing of ZNF8 downregulates naïve hESC marker expression and impairs blastoid formation capacity. (**a**, **b**) Immunostaining (a) and quantification (b) of DNMT3L and TFCP2L1 expression in *ZNF8-IF* and NT naïve hESCs. (**c, d**) PCA plot (c) and distance heatmap (d) of *ZNF8-IF* and NT naïve hESCs. (**e**) Heatmap of naïve pluripotency marker expression in *ZNF8-IF* and NT naïve hESCs. (**f, g**) Immunostaining (f) and quantification (g) of CDX2 and GATA3 expression in *ZNF8-IF* and NT naïve hESC-derived TSCs. (**h**) Flow cytometry analysis of CDX2 and GATA3 expression in *ZNF8-IF* and NT naïve hESC-derived TSCs. (**i**) qRT-PCR analysis of *CDX2*, *ELF5* and *TFAP2C* expression in *ZNF8-IF* and NT naïve hESC-derived TSCs. (**j, k**) Immunostaining image (j) and quantification (k) of the percentage of OCT4+ and GATA3+ cells of NT naïve hESC-derived blastoids and *ZNF8-IF* naïve hESC-derived cell clusters. Data represent the mean ± s.d. *P* values of figures were calculated by one-way ANOVA, *P<0.05, **P<0.01, ***P<0.001, ****P<0.0001, ns, a non-significant difference.

ZNF8 is a Krüppel-type zinc finger protein, which is predicted to bind DNA^16^. To further investigate the role of ZNF8, we first examined the expression of naïve hESC markers and found that *ZNF8-IF* naïve hESCs lose DNMT3L expression and show significantly reduced expression of TFCP2L1 (Fig. 4a, b). Next, we used RNA-seq to compare the *ZNF8-IF* and NT naïve hESCs. A PCA plot and distance heatmap revealed distinct transcriptome profiles between *ZNF8*-*IF* cells and NT naïve hESCs (Fig. 4c, d), with the decreased expression of naïve pluripotency marker genes in *ZNF8-IF* cells (Fig. 4e). One unique feature of naïve hESCs is the ability to differentiate into the trophoblast lineage ^17^. *ZNF8-IF* naïve hESCs failed to differentiate into trophoblast stem cells (TSCs), as indicated by the significantly reduced expression of the TSC markers, CDX2 and GATA3 (shown by immunostaining, Fig. 4f, g, and flow cytometry, Fig. 4h, Extended Data Fig. 6b) and *CDX2*, *ELF5*, and *TFAP2C* (shown by qRT-PCR, Fig. 4i).

Recently, blastoids have been derived as *in vitro* models of human blastocysts, and naïve hESCs, competent to differentiate into both embryonic and extra-embryonic lineages, are usually used to derive blastoids ^18–21^. We asked if we could induce blastoid formation from *ZNF8-IF* and NT naïve hESCs. We successfully derived blastoids in NT naïve hESCs that showed a blastocyst-like structure which contains OCT4+ internal cell mass-like cells and GATA3+ trophectoderm-like cells (Fig. 4j). However, we failed to detect a similar blastoid-like structure in *ZNF8-IF* naïve hESCs-derived cell clusters. Both the percentage of OCT4+ cells and GATA3+ cells are significantly lowered in the *ZNF8-IF* naïve hESC-derived cell clusters compared to NT naïve hESC-derived blastoids (Fig. 4k). Together, it suggests the ZNF8 might play a critical role in blastoid formation.

## Discussion

Recent advances in sequencing technologies to analyze chromatin accessibility across the genome, such as ATAC-seq, have been used to identify specific DNA regions associated with certain cell types or disease states. Although methods have been developed to identify the downstream genes regulated by specific DNA regions, there are very few tools to identify the upstream protein complexes that bind with specific sites in the genome (such as promoter or enhancer region) and regulate gene expression. Here, we developed the SUPER method by incorporating the photo-crosslinking ncAA AbK into dCas9 and subsequently used it to generate hESCs^SUPER^ to examine DNA-protein interactions in a cell-context and genomic site-specific fashion. There are three major advantages of the SUPER system. First, SUPER uses AbK, a photo-crosslinking ncAA that captures proximal molecules through covalent bonds upon UV activation, which stabilizes protein complexes to isolation conditions much more than non-covalent biotinylation systems ^22–26^. Unlike biotinylation, which can non-specifically interact with other biotin-binding proteins or cellular structures causing off-target interactions and background noise, the highly sensitive AbK system improves the signal to noise ratio, enabling the capture of dynamics DNA-protein interactions. Secondly, the dCas9 system can theoretically direct AbK to any specific genomic region with the appropriate sgRNA. Finally, the engineered hESC^SUPER^ line can differentiate into various cell types, enabling investigation of cell-context dependent DNA-protein interactions.

To evaluate the SUPER methodology, we first designed sgRNAs targeting the *NANOG* promoter, which is a known OCT4 binding site. The SUPER system not only enriched the known binding proteins, OCT4 and SOX2, validating the system, but also identified previously unknown protein, CARHSP1, which binds the *NANOG* promoter region at the hPSC stage. CARHSP1 has been reported to be involved in zygotic genome activation during early embryonic development ^27^. We found that at the hPSC stage, an OCT4/SOX2/CARHSP1 protein complex binds to the *NANOG* promoter region, activating NANOG expression and maintaining pluripotency. By comparing protein DNA interactions at the hPSC and ectoderm stages, we identified ZIC2 as a repressor that binds the *NANOG* promoter region to inhibit *NANOG* expression. ZIC2 is known to regulate the transition from both primed ^28^ and naive pluripotency ^29,30^ and serves as a master regulator of neurogenesis ^31–33^. Additionally, ZIC2 functions as a cofactor with Mesp1 to control mesoderm specification ^34,35^. However, the precise mechanisms by which ZIC2 regulates pluripotency remain unclear. Using the SUPER system, we found that, upon ectoderm differentiation, ZIC2 acts as a competitive repressor, occupying the NANOG promoter region, suppressing NANOG expression, and initiating ectoderm differentiation.

Next, we used the SUPER methodology to explore the distal enhancer region of *OCT4* which is highly active in naïve, but not primed hESCs. SUPER identified ZNF8, a zinc finger protein, that binds the *OCT4* distal enhancer region. Until now, very few functions of ZNF8 has been reported, including regulating BMP signaling ^36^ and controlling the progression of gastrointestinal ^37^ and breast cancer ^38^. Here, we found that ZNF8 binds the *OCT4* distal enhancer region and maintains naïve pluripotency status. Silencing of ZNF8 results in decreased naïve marker expression and impaired ability to form blastoids.

In conclusion, we have developed a highly sensitive SUPER methodology to map cell context-dependent, site-specific DNA-protein interactions. The SUPER system enables selective identification of protein regulators binding to specific genomic regions, making it a powerful tool for studying regulatory mechanisms throughout cell fate transition. This system can also be applied to investigate DNA-protein interactions in various physiological and disease contexts, providing valuable insights into the molecular underpinnings of cellular function and dysfunction.

## Methods

### Plasmid construction

The origin amber suppression plasmid pEBV1 has been published previously^8^. The six copies of PyltRNA were PCR amplified from pEBV1 plasmid and digested with SpeI and SphI restriction enzyme, and subsequently ligated into the backbone plasmid pAAVS1 containing a CAG promoter (Addgene, 80490). Then, the IRES-MbPylRS construct was PCR amplified and inserted into the previous plasmid after EcoRI + PacI dual digestion. The amber-containing dCas9 mutant was generated by site-directed mutagenesis using high fidelity PCR enzyme and inserted into the upsteam of IRES-MbPylRS using NotI and AscI.

### hPSC culture and differentiation

All hPSC work was approved by the TRI-SCI ESCRO committee. hPSCs were cultured on Matrigel-coated plates with StemFlex medium (Thermo Fisher) and maintained at 37 °C with 5% CO2. Naive hPSCs were cultured in the 5i/L/A media as previously described^39^. The5i/L/A medium is prepared as follows: 1:1 mixture of Neurobasal and DMEM/F12 supplemented with 1X N-2 supplement, 1X B-27 supplement, 2 mM GlutaMAX, 1X MEM nonessential amino acids, 50 mg/L bovine serum albumin fraction V (BSA), and 0.1 mM 2-mercaptoethanol, 1 μM PD0325901, 1 μM IM-12, 0.5 μM SB590885, 1 μM WH4–023, 10 μM Y-27632, 10 ng/mL Activin A, and 20 ng/mL recombinant human LIF. Media is stored at 4 °C for up to 2 weeks. Naive cells were induced by changing to the above medium every day and passaged every 5–6 days.

For the differentiation to three germ layers, endoderm was induced with Activin A (R&D systems) and CHIR99021 (Cayman Chemical) according to the previously published protocol^40^. In brief, hPSCs were digested to single cells using Accutase (Thermo Scientific) and reseed onto Matrigel coated 6-well plate. Once the hPSCs reached 80% confluence, the media was changed to RPMI 1640 supplemented with 100 ng/ml Activin A and 3 μM CHIR-99021 to initiate the differentiation. 24 hours later, the medium was switched to RMPI 1640 plus 100 ng/ml Activin A and 0.2% FBS and maintained for 48 hours. Then, cells were collected for qRT-PCR and immunostaining. The mesoderm differentiation was induced with RPMI 1640 supplemented with B27 minus insulin + high concentration of CHIR99021 (6 μM for H9 and 9 μM for H1 hESC) for 24 hours, followed by RPMI 1640 supplemented with B27 minus insulin. Cells were collected at day 4 for qRT-PCR and immunostaining. The ectoderm differentiation was induced with 10 μM SB431542 (Cayman Chemical) and 500 nM LDN193189 (Stemcell Technology) in DMEM/F12 medium supplemented in N2 (Thermo Scientific) and B27 (Thermo Scientific) for 48 hours to 72 hours with daily change of fresh medium. Cells were collected at day 4 for qRT-PCR and immunostaining.

Trophectoderm stem cell (TSC) differentiation was induced according to published protocol^17^. Naive cells were dissociated into single cells and transferred to a 15 mL Falcon tube with 5 mL hTSC medium, including DMEM/F12 supplemented with 0.1 mM 2-mercaptoethanol, 0.2 % FBS, 0.3 % BSA, 1 % ITS-X, 1.5 μg/mL L-ascorbic acid, 50 ng/mL EGF, 2 μM CHIR99021, 0.5 μM A83–01, 1 μM SB431542, 0.8 mM VPA, and 5 μM Y-27632. After centrifugation at 250 g for 3 mins, the cells were resuspended with fresh hTSC medium, and seeded at 0.5–1.0×106 naïve hESCs per well in a 6-well plate. The media is replaced every 2 days with 2 mL fresh hTSC medium until the cells reach 80–100 % confluence.

Blastoid formation was conducted following the published protocols^18,40^. Naïve hPSCs cultured in the 5iLA condition were dissociated into single cells by using Accutase (Thermo Scientific). An AggreWell-400 (STEMCELL Technologies) plate was prepared by adding 500μL of anti-adherence solution (STEMCELL Technologies) to each well, followed by centrifuge at 1500 × g for 5 min, and then incubated at room temperature for a minimum of 45 mins. Wells were then washed with eHDM once, and 0.5 ml fresh eHDM was added. Approximately 30000 cells (25 cells per microwell) were resuspended in 1 mL eHDM and seeded into one well of a prepared AggreWell-400 24-well plate. After 24 hours, 1 mL of old medium was removed and replaced with 1 mL of fresh eHDM medium. On day 3, as much eHDM as possible was carefully removed without disturbing the aggregates. For the following days, half of the medium was replaced daily with fresh eTDM containing freshly added LPA.

### Transfections and photo-crosslinking

H9 hESCs or H9 naïve hESCs were transfected with the SUPER plasmid and sgRNA containing plasmid using PEI method. The culture medium was changed to freshly made StemFlex or 5i/L/A media supplemented with 1 mM AbK or 1 mM BocK at 4 hours after transfection. After 48 hours, the medium was replaced with PBS and cells were exposed to 365 nm UV light on ice. The harvested cells were then immediately stored at −80 degree prior to lysis and affinity purification.

### Enrichment of photo-crosslinking proteins

Cell pellets were resuspended in 50 mM Tris (pH 7.4), 150 mM NaCl, and complete EDTA-free protease inhibitors, then incubated for 10 mins at 4°C. The suspension was sonicated to disrupt the cell membrane and chromatin (5 secs on and 5 secs off for 60 cycles) and then centrifuge at 12,000 g for 10 mins at 4°C. The supernatant was incubated with M2 FLAG affinity magnetic beads at 4°C overnight with gentle rotation. The beads were washed three times with ice-cold wash buffer (50 mM Tris-HCl, pH 7.4, 150 mM NaCl, complete EDTA-free protease inhibitors). The beads were then eluted with acid elution buffer (0.1 M glycine, pH 3.0) and neutralized with 1.0 M Tris-HCl, pH8.5.

### Mass spectrometry analysis

The protein samples were acetone precipitated and re-suspended in 0.1% RapiGest (Waters), 25 mM ammonium bicarbonate. The samples were then reduced with DTT, alkylated with iodoacetamide, and digested overnight with trypsin at 37 °C. The digests were desalted by C18 Stage-tip columns.

The digests were analyzed using a Thermo Fisher Scientific EASY-nLC 1200 coupled on-line to a Fusion Lumos mass spectrometer (Thermo Fisher Scientific). Buffer A (0.1% FA in water) and buffer B (0.1% FA in 80 % ACN) were used as mobile phases for gradient separation. A 75 μm × 15 cm chromatography column (ReproSil-Pur C18-AQ, 3 μm, Dr. Maisch GmbH, German) was packed in-house for peptide separation. Peptides were separated with a gradient of 5–40% buffer B over 30 min, 40%−100% B over 10 min at a flow rate of 400 nL/min. The Fusion Lumos mass spectrometer was operated in a data independent acquisition (DIA) mode. MS1 scans were collected in the Orbitrap mass analyzer from 350–1400 m/z at 120K resolutions. The instrument was set to select precursors in 45 × 14 m/z wide windows with 1 m/z overlap from 350–975 m/z for HCD fragmentation. The MS/MS scans were collected in the orbitrap at 15K resolution. Data were searched against the human Uniprot database (8/7/2021) using DIA-NN v1.8^41^ and filtered for 1% false discovery rate for both protein and peptide identifications. The mass data was processed using the R DEP package^42^.

### Generation of inducible target gene knockdown cell lines

An inducible H1 dCas9-KRAB ESC line was generated as previously reported^43^ and employed for targeted knockdown experiments. Briefly, a H1 ESC line which has an inducible Cas9 cassette located at the AAVS1 locus was edited by targeting the Cas9 protein to the AAVS1 DNA flanking regions and providing a dCAS9-KRAB donor plasmid with a Blasticidin constitutive cassette. hESCs were treated with doxycycline (2 μg/ul) for 2 days to activate the expression of Cas9 and transfected with the gRNA targeting the AAVS1 flanking regions and the dCas9-KRAB plasmid containing the DNA homology arms to target the AAVS1 locus. After, cells were selected with G418 (500 μg/ml) and Blasticidin (10 μg/ml) to select the clones with the dCas9-KRAB cassette. Individual clones were genotyped and tested for dCas9-KRAB expression and repression activity, and the clone with the highest expression was selected for further experiments.

The sgRNA plasmids were transfected into 293T cells together with pMD2.G and pspAX2 using Calcium Phosphate Transfection to produce lentivirus. After collecting lentivirus and concentrating with Lenti-X Concentrator (Clonetech), the inducible H1 dCas9-KRAB line was infected and selected with puromycin after 48 hours. After 72 hours treatment with 1 μg/ml doxycycline, the cells were collected for RNA extraction or immunostaining. The sequences of sgRNAs are provided in Table S1.

### Perturb-Seq

We first incorporated a random 22 nucleotide genetic barcodes (GBC) into the backbone plasmid pPS. The pooled sgRNA mixture was PCR amplified and ligated into this plasmid. The sequences of sgRNAs are provided in Table S2. The sgRNA-GBC correlation reference table was generated by sequencing. Next, we cloned an EGFP coding sequence along with the EF1α promoter and inserted it between the sgRNA and GBC elements. The final construct was packaged into lentivirus in 293T cells. A separated plasmid containing the CRIPSR/Cas9 coding gene and co-expressed mCherry was packaged into lentivirus. The hPSC cells were infected with lentivirus and sorted based on fluorescence protein expression. The sorted cells were recovered for one week, followed by induced differentiation towards ectoderm lineage. Ectoderm cells were collected and prepared for single cell RNA-seq according to the 10x Genomics Single Cell Protocols Cell Preparation Guide (10x Genomics). Cells were separated into droplet emulsions using the Chromium Controller (10x Genomics) with Chromium Single-Cell 30 Gel Beads v3 (10x Genomics) following the 10x Genomics Chromium Single Cell 3ʹ Reagent Kits v3 User Guide with the goal of recovering 10,000 cells per GEM group before filtering.

To generate the correlation table between cell barcodes (CBC) and GBC, we conducted another round of enrichment PCR and sequencing using intermediate product from the previous library preparation. For perturbation effect analysis, each cell was first annotated with its perturbation target by referring the GBC-sgRNA reference table and CBC-GBC correlation table. Cells perturbed with more than one target were excluded from the analysis. We then compared gene expression and pseudotime by grouping cells according to their specific perturbation targets.

### Single-cell RNA sequencing data processing

The 10x libraries were sequenced on the Illumina NovaSeq6000 sequencer with pair-end reads (28 bp for read 1 and 91 bp for read 2). The sequencing data were primarily analyzed by the 10x cellranger pipeline (v7.0.1) in two steps. In the first step, cellranger mkfastq demultiplexed samples and generated fastq files; and in the second step, cellranger count aligned fastq files to the reference human genome (Human GRCh38 (GENCODE v32/Ensembl98)) and extracted gene expression UMI counts matrix.

For filtering, we excluded dead cells and doublets by selecting the cells with more than 500 but less than 5000 detected genes, and with no more than 10% mitochondrial genes. The raw gene counts were normalized and scaled by the function provided by Seurat R package (version 4.0)^44^. Distances between cells were calculated with the FindNeighbors function, followed by clustering with FindClusters function (with a resolution of 0.2), both from the same package. Dimensional reduction was performed using the t-distributed stochastic neighbor embedding (t-SNE) algorithm, and final cluster annotations were based on marker gene expression.

The trajectory analysis was conducted by using R package Monocle 3 (version 1.2)^45^. Specifically, the Seurat object was converted to celldataset object for Monocle3, and clustering information from Surat object was retrieved. The trajectory was learned and constructed using the learn_graph function, with clusters colored according to pseudotime after setting the root cell population. The plot_genes_in_pseudotime function generated the expression patterns of selected gene along pseudotime. To demonstrate the pattern along pseudotime, perturbation and marker genes for PSC and ectoderm lineages were combined into a heatmap using the ComplexHeatmap R package^46^.

### Bulk RNA-seq and data analysis

RNAseq libraries of polyadenylated RNA were prepared using the TruSeq Stranded mRNA Library Prep Kit (Illumina) according to the manufacturer’s instructions and sequenced on an Illumina NovaSeq6000 platform. The resulting paired-end reads were processed using the custom workflow. Specifically, the raw reads were mapped to human reference genome GRCh38 using HISAT2 aligner^47^ and the gene counts were calculated with featureCounts function^48^. The resulting counts table was imported into R along with the sample information table. PCA was conducted with prcomp function from R. and the plots, including PCA and heatmap, were generated by using ggplot2 package.

### qRT-PCR

Total RNA from each sample was extracted and purified with RNeasy Plus Kit (Qiagen) and reverse transcribed with high-capacity cDNA reverse transcription kit supplemented with RNase inhibitor (Thermo Fisher). qPCR reactions were performed using SYBR green gene expression 2X Mix (Vazyme). Expression levels were quantified relative to the human housekeeping gene TBP, which was used as an internal reference. Primer sequences are provided in Table S3.

### Immunostaining

Cells were directly fixed in 4% paraformaldehyde for 30 min, followed with 60 min permeabilization in 0.1% Triton X-100. For immunofluorescence, cells were immuno-stained with primary antibodies at 4°C overnight and secondary antibodies at RT for 1 hour. The information for primary antibodies and secondary antibodies is provided in Table S4. Nuclei were counterstained by DAPI. For the blastoids, the fixation was conducted in 4% paraformaldehyde for 30 min. Then the blastoids were transferred to the tubes containing fresh PBS to wash for three times, followed by overnight permeabilization in 0.1% Triton X-100 at 4°C. The primary antibodies and secondary antibodies stained for 48 hours at 4°C.

### Quantification and statistical analysis

N=3 independent biological replicates were used for all experiments unless otherwise indicated. P-values were calculated by one-way analysis of variance (ANOVA) unless otherwise indicated. *P<0.05, **P<0.01, ***P<0.001, ****P<0.0001, ns, a non-significant difference.

## Extended Data

## Supplementary Material

Supplement 1

## Figures and Tables

**Table 1.** Sequences of sgRNAs used in SUPER system.

Target	Sequence	
*NANOG* promoter	TAGGGGGTGTGCCCGCCAGG	sgRNA_1
	TTCACAAGGGTGGGTCAGTA	sgRNA_2
	AATCAGGAATATGGTTCAAC	sgRNA_3
*TBP* promoter	GAGCAACTCGCTGAAGCCGA	
*OCT4* distal enhancer	GCTTGGGAAAGAGCGCTTTTG	

## Data Availability

All data are available in the main text or the supplementary materials. The raw sequence data are uploaded to the GEO database. https://www.ncbi.nlm.nih.gov/geo/query/acc.cgi?acc=GSE283073 for bulk RNA sequencing and https://www.ncbi.nlm.nih.gov/geo/query/acc.cgi?acc=GSE283074 for single cell RNA sequencing).
