# Supplementary material for: A Chemical Epigenetic Probe to Capture the Site-Specific DNA-Binding Protein Complex
Source: Res Sq. 2025 Mar 11:rs.3.rs-5915426. Preprint. [Version 1] doi: 10.21203/rs.3.rs-5915426/v1 (PMC11952667; doi:10.21203/rs.3.rs-5915426/v1)
Supplement: Supplement 1 [file NIHPPRS5915426V1-supplement-1.pdf]

Table S1. List of sgRNAs for Gene Repression

Target Gene	sgRNA	Sequence
CARHSP1	IF_1	GCGGAGCAGAACGGATTGCA
	IF_2	CGCTCTCAGTCGGAGCGAAG
ZIC2	IF_1	GTATTAAAGGAGCTGCGGCG
	IF_2	ACACTCACTCGCACGCCCAG
ZNF8	IF_1	CCGGCGTTCGGCGAGTCGGG
	IF_2	AGGTGATCCCCAGACCAGAG
NT	NT_1	GCGGGCAGAACGACCCTGAC
	NT_2	GTAGGCGCGCCGCTCTCTAC

Table S2. List of sgRNAs Perturb-Seq.

Target Gene	sgRNA	Sequence
ACAD8	1	CAGGCCTTGCAGATGCACGG
	2	AACTTCTCCATGGTACAGAG
	3	GCTTCGGAAATGAGGAACAG
	4	CTTAGCGGAGGTCAGAAGAG
ACOT13	1	GCAATAGGCACTCTCCACGG
	2	ACGTGGCTGTCAAACCGCCG
	3	GGTGATAAAGGCCATGACCA
	4	CCAGCATGACTCAGTCTCTG
ACOT9	1	TTTTACCCTCTAATGCAGTG
	2	GAACAGGACATTAAGTTCAG
	3	AGTCAGAGTACACAGTGAAG
	4	GGAAGGCATATGAACTTGCG
AHCTF1	1	TCTGCCATACCTAGAAAACG
	2	ATCCATTTTAGTGCCAAACG
	3	TGTCTGCTCCTTAAAGCAGG
	4	AGAATTGAATCACTTAAGCC
ALDH4A1	1	GGAACCAATGACAAGCCAGG
	2	GTCCAGCGCAGGATGTAGTG
	3	GCGCCCTGCTGTCCCGCCCC
	4	CGGTCCAGGGGCGGGACAGC
ALDH6A1	1	ATGCCTGCACACACTCCCAG
	2	GAAGGTATTTCCACACACCA
	3	CATCTGGCATGACTACCCCA
	4	GGAAAATACCCTGAACCAGC
ARG2	1	CATCAACCCAGACAACACAA
	2	TACAGCTGTGTCACACTGGG
	3	CCTTATCCTGTAGTTCTCTG
	4	ATACTTGCAGAAGAGATACA
ARL6IP1	1	AGTACATTGGAATGGCCAAG
	2	CTTCCTGGACTAAACCAACA
	3	AAGTTTGTTTATCTCCCTCT

	4	ATTCCATGTTGGTTTAGTCC
AUH	1	AAATTGGCGATTATTCCTGG
	2	ATAGATGGACTCGCTTTAGG
	3	AAAATGAGTTCCAGTGAAGT
	4	GATGGACTCGCTTTAGGTGG
BMS1	1	TATTATATCGAAGACCACAA
	2	GAAGCCTTTGTCTTCCATTG
	3	AAAGCACTCCGAGCTCCAGA
	4	GCTGTGATTCGAACAGTCAG
BUD13	1	GGTGCTGGACCACTGTAGCG
	2	CAGATATTAAATCTGTTGGG
	3	TCATCAATATAGCGGGCCAG
	4	GAGATGAGCTGTATGCCCAG
C1D	1	TGAGAATTCCATTGGTGCTG
	2	CATCTCATCCACAGCACCAA
	3	TCCACTTGAACAAGCAAAAG
	4	AACCCAAGGAGTTAATCCTA
CCDC134	1	TGCAGAAACTGATACCCCAG
	2	CAGACAAAAGCAGGACAAAG
	3	ATCTGGGAGCACATCAGCAG
	4	TGATACCCCAGCGGATGAGG
CCDC58	1	AGTACATGAATTAAACACTA
	2	AGAACTGAATGTTGAAGAAG
	3	AAGTTGTTTACAGGTTTGGC
	4	AAGTGGTAAATGACAGGAGC
CDH1	1	GTGAAGGGAGATGTATTGGG
	2	TCAATGATGTTTATGACCTG
	3	ACACACGCTGACCTCTAAGG
	4	CAACCCTCATGAGTGTCCCC
CHID1	1	GCTGAAACCATCCAGCACGG
	2	TAGAGTAGTCGTAGGTCATG
	3	TATGGACTACGCGACCTCCA
	4	AGGCTCACGGGCATCCTTGG

CNP	1	CAAGAATTCTGATGTCCCGG
	2	CGTCTTGCACTCTAGCAGCG
	3	GTGCAATGCAGCACGCCTGG
	4	CAAGTTTTGTGACTACGGGA
CPOX	1	TAGCCCATCGTGGAGAACGG
	2	TCTTGACTCTCCGTCCAAGG
	3	GATTCAAGTATGTTGGAGTG
	4	TTTTCACAGAACTCTGAAGG
CPSF2	1	ATTCAAGCTGAATTTGTAGG
	2	CATCGTCCATGGCCCACCAG
	3	CAGTTTTTATGAATGAACCA
	4	CGACAGTTGATCATCGTCCA
CPSF3	1	GCAGTGACCTTAAACTTCAG
	2	AATACAAGAACCAGGCATGG
	3	CTCTATTCTTAGCGTCACAG
	4	CCATTTCATTCTGTTCTCCA
DDX24	1	TCGGTGGACATAAATCTCCG
	2	GAGCAGTTCGACCACTTCGG
	3	CTGATGCACCAGGGTGAGTG
	4	GGTCAGCCTCATCCACTACC
DHRS4	1	TCTTGATAAGTCCAGGTGCT
	2	AGGGTGAACTGCCTAGCACC
	3	CGCCTTCCCCACATGGCACA
	4	GCCCGGCGTTTGGCCCAGGA
DHTKD1	1	GAAACAAAGAGAATCTCTGG
	2	AACCTCAGAACATGGGTCCG
	3	CCATCTGTCTCCTAAGCAAG
	4	TTAGGGGAAGCAACAATGAG
DNASE2	1	GAGGGAAGTTAGGTACACTG
	2	TATCTGGACGAGAGCTCCGG
	3	TATGCAGCAGAGGAGGCCGG
	4	GTAGTTATAGACCCAGGGGT
GLA	1	GAGCAGCCATGATAGCCCAG

	2	GCATTGTGTACTCCTGTGAG
	3	GCTAGCTGGCGAATCCCATG
	4	GCTCCCCAAAGAGATTCAGA
GLIPR2	1	CTGCAAGAACCTCAACCGGG
	2	AATGAGTACCGGCAGAAGCA
	3	GATCATAGGATGCCCATGCA
	4	GAGGATCCTCAAGCACAGCC
GLS	1	CTGTTGCCCATCTTATCCAG
	2	TTGCCCATCTTATCCAGAGG
	3	CCAGAAGGCACAGACATGGT
	4	CAGTTTTTGAATAAGATGGC
GNL2	1	CTTACCCACAGTCTGCAAGT
	2	AGAGCCCGACTTGCAGACTG
	3	TCAGAGGGGTAAACCACACC
	4	GTCTGTCTCGGAGTCCTCAG
GPT2	1	TTACAGAGGAGGCTACATGG
	2	GTTCACGACAATGTCCATGG
	3	GAGAAAGAGCTTCTCTTCCC
	4	GGAAGAGAAGCTCTTTCTCC
GTF2F1	1	GAAGAAGGGTTCAGACGACG
	2	TGGGGACTTCGAGGGCCAAG
	3	GCGAGATGCCTGCAGCCAAG
	4	GGTGTTGTCTTGCCTGATGG
HACL1	1	TGGGCAATGGATCATCTGTG
	2	ATCCTCCAGAGAGCAAGTGG
	3	AGTATCCTCCAGAGAGCAAG
	4	GCTTCTATCTTTAGCCACCA
HIST1H1T	1	TGTGTCCAAGTTGATCACCG
	2	TGATCAACTTGGACACAGAG
	3	TCAGTGTCACAGGAACGAGT
	4	TCTTGAGGGACAGTTTGATG
HMGA2	1	ACTGGAGAAAAACGGCCAAG
	2	AACGGCCAAGAGGCAGACCT

	3	TTAGGAGAGGGCTCACCGGT
	4	TCCCCTGGGTCTCTTAGGAG
JMJD1C	1	GAACGGCAGAAAAACAAGAG
	2	TGAACAAAAAGCTCCGTCAA
	3	TGATGCTATTGTTTTGCCAG
	4	GAATTTTCACAGCTGTATTC
LAMB1	1	CAGCGAGTTAGAGAGGAATG
	2	AGCTGCCCAAAACTCCGGGG
	3	AAGAGTCAGCTGATGCCAGA
	4	TGAAAGCCTTTCTCAAGTAG
MANBA	1	CTATCTCGCTGCGACTACGG
	2	CCACTTCTTGTCCTCACCGA
	3	TCAAAGCCTACTGGCAACAG
	4	GAACGTTTTGTGATGAAAGG
MANF	1	AGCCACCAAAATCATCAATG
	2	TTTGGTGGCTGCATCATCTG
	3	GTGGGCCACGCAGGGGCTGG
	4	TGAGCGTGCTGCCGGGCAGC
MDC1	1	ACAGGAACTTGACTGTCCGG
	2	GTGGTCAGAGCAAAACACTG
	3	GTCACCACATATTCATCCGG
	4	AACCGGCATAGCTCTTACTG
MRPL11	1	GGAGTTCAATGAGAGGACAA
	2	ACACATGCTTCAAGGTCACC
	3	CTTCAGGAAGTAGGAAACAG
	4	TTTGCAAGGAGTTCAATGAG
MRPL14	1	CAGATTCGACTCCAACAACG
	2	TTATAGACATGGATGCAGCG
	3	GCTCAGCACTCTGCTTACAC
	4	CACTCTGCTTACACAGGTGA
MRPL3	1	ATGGTCAAACGAAAACCCAC
	2	AAAGGCCAGCCTGCTACGCA
	3	TGGCCTGGAACTAAAATGCC

	4	GTGACATCCACATACTGTCC
MRPL33	1	AATACGCTCCCTTTAAACGG
	2	ACACCAAGAGAAACCGACTG
	3	GATTTATAAAGAGAAGACTG
	4	GAGTCAGTTTTTCCCGCAGT
MRPL34	1	ATCAAACGCAAGAACAAGCA
	2	GCGAAGGATGACCTGCACGC
	3	GTTGGGCCCCACGAGTAGGT
	4	CAACGCTGCCGACCTACTCG
MRPL46	1	TCTCTCTGACTAACAGGACA
	2	AGGAACAGCTGAACGAACCC
	3	GAAGCTCGTGGTCTGAATAC
	4	AAATTTCTACAGTTCAAACT
MRPS12	1	GTGCGTGGCAAGTACGACTG
	2	GTCGTACTTGCCACGCACAA
	3	CAGATTGTCCTTGTGGAGGG
	4	GTCAAGCTCACCGTTGTGCG
MRRF	1	ATCCAAGGATACAGTCTCAG
	2	ATATTCACCAAAATCAGCTG
	3	ATCCCTTGACAAGATTGCTG
	4	GAACAAGCTGAAGAAATCCA
MTA2	1	ATCGGTGTCTTGGTACCCCG
	2	GAACGAATCCCTGAAGCCAG
	3	GATAAATGACCCCTTGAGTG
	4	GGGACCTATTACAGCCAAGG
MTCH1	1	ACAAGAAAACCACATCGCCC
	2	CTCTTTGAAAATCTTCCCAA
	3	CCCTCACCTCCTGGGCGATG
	4	GGGAAGATTTTCAAAGAGGA
NOP14	1	CTTGGACCTGGAATCCAACG
	2	CGTTGGATTCCAGGTCCAAG
	3	GGCCGTTCGAGAAATCCGCA
	4	TTGCTCTTCTCACAGGTCAG

NSUN4	1	GGCCAGAACAGGCAGCAAGG
	2	AGACCGCCACTCCCTTCATG
	3	CCATTTCCTGCCATCCCATG
	4	CCTCCTCATGAAGGGAGTGG
NUCB1	1	AGTTCCTCGCATCCACTCAG
	2	ACTCCTCCAGGGTCACGAGG
	3	GTACTGGGCAAGGTCCCGGG
	4	CAGCCAAGCCCAGTTGAAGG
NUP98	1	ACCTGCCAAATGGATCCACG
	2	AGTGTCGGATGATGAGCTTG
	3	GCTGAGCGCCCTAGAAATGG
	4	ATTAGGCTGCTGCGCTCTGG
OXCT1	1	GTCCAGTGCGAAAACCAAAG
	2	CAGTGCGAAAACCAAAGTGG
	3	CACGTCGATCTGACAATGCT
	4	TTTGATGTGGACAAGAAGAA
PATZ1	1	GTCCATGTTAAAACCCACCA
	2	CTAGGAAGAGGTTCCAGGGG
	3	CTTGAACGGACATATCAAGC
	4	ATATGTCCGTTCAAGTGATC
PCCA	1	TTCCCCGATGCCCGGAGTGG
	2	GTTCTGCGTTCCCCGATGCC
	3	ATGCAGAATAGTATGACAGC
	4	AGTATGACAGCTGGGAAAAC
PCK2	1	GGGGAGGGAGAACAGCTGAG
	2	GAGTGGTGTCTATAGCTCTG
	3	AGGCCGCATCATAGCCAGGT
	4	AGACCAACCTGGCTATGATG
PDF	1	CTGCGTGCCCCGCTTCCAGG
	2	GGTTCACGAACACGCGCAGG
	3	CCGCCTGGTCACCTTTCCCG
	4	GTCGGGGACCCGGTGCTGCG
PMPCA	1	AGCAGACACAGCATACATGG

	2	AGGTCGCGTTATACATCCAG
	3	GTGTTGAACATGATGATGGG
	4	TGATAGCAAAGGCTTGGACA
PPIF	1	TAAAGTTCTCGTCAGGAAAG
	2	GAACTTTACACTGAAGCACG
	3	GAGAAGGGCTTCGGCTACAA
	4	TTATGGTGCAGATGAAGAAC
RANBP10	1	GGTGAATGGGACGGACAGTG
	2	TCACCATCTCCACAAACTGC
	3	CTGTTGAGGGCAGCACACAC
	4	GGAGTCTGTTGAGTTGGACT
RPS19BP1	1	GACCAGGACGAGAAGCACCG
	2	CTGGCTCACAGACTCAGCCA
	3	CCAGGTCAGGCCAAGCCGAG
	4	CAAGGGAAAGGTGCCCAAGT
SALL4	1	GCGCTCTTCAGATCCACGAG
	2	ACCACAGACAGAGCACACGA
	3	CTCGGCCACAGATCTTACAC
	4	CCAAGCAACATGGCTGCACA
SCO1	1	ATCGGCAAGCCTTTACTTGG
	2	TGGGGAGCGTAAAACTGACA
	3	GTATTACAGCCCTGGCCCCA
	4	GAGAGAAGAGGTCGATCAAG
SOX2	1	CTCCGACAAAAGTTTCCACT
	2	TTTTGTCGGAGACGGAGAAG
	3	ACAGCCCGGACCGCGTCAAG
	4	GTGGAAACTTTTGTCGGAGA
SRFBP1	1	ATGTCTGAAGATAGTGATAG
	2	CTCTCTGGTAACAGTGATGG
	3	CACTGTTAAAAAACCAAAGA
	4	ATAGTAACTAAATCTGCTCT
STRBP	1	GAGCTCAATGAAAAAAGAAG
	2	AAGTGGCAAAAACCCTGTAA

	3	GCAGCTGTGCTGTAACCATA
	4	GCTGTTCGGGGCAGAGGAAG
SURF4	1	GGTACTGCTTTGTGACACGG
	2	TCTGATCAGCACCTTCCTGG
	3	CAAGTCCCATAAAATGCTGT
	4	ACATGCTCTTCCCTTCAGAA
THOC1	1	GAGCCCTGAGACTATTAGCA
	2	CTATCTGAAAACCCCCCCGA
	3	GGCAGACCCTGAAAATATGG
	4	TTGGTTGGCTGGAAGAAGTG
THOC3	1	CTAACTCATCCACATCCCAG
	2	CAAAGCATCTGCACTTCCTG
	3	CTCGGGGTCCTTCGACAAGA
	4	GGAACTCGCGCGTCTTGCTG
THOC5	1	GCCAGGGTTCAACACCACAG
	2	TCATGATGTTGAGGTTCATG
	3	CGAGAGCCATGACGACAGTG
	4	ACACAGCCGCTGCAGCTGGT
TIMM10	1	GAGGAGGCACACACTTCCGG
	2	AGTACCTGGACATCCATGAG
	3	GCCAGCTGTTGGGCCCTGAG
	4	TCCTCTCAGGGCCCAACAGC
TP53I11	1	GACCCGCAAGATCCTCGGCG
	2	ACCCCCATCCGCCTCTACGG
	3	GAGCCCCAAAGGCTCCCGAA
	4	GATGAAGAAGCACAGCCAGA
TSFM	1	TGGAAGTCCAGAAAGCTCAG
	2	TGACAGAGAAGGCTCACTCA
	3	GAGACAGCTCCAATTTGGAA
	4	TTCTGCAAGAAGTTGGATTC
TST	1	TCTGTTCCAGACCAAGAAGG
	2	AAGGCATGTTGACGGCACCA
	3	CATCAGCAACCACACGCACG

	4	TGGATTCAAGGTCTCAAGGG
UTP14A	1	AACTGCCAAAGACTTCACGG
	2	AGCCTTAAGCACCAAAACAG
	3	TGTGGAGGCGAGTAAGCCAA
	4	GTTTCTCATTAAAGCCCCTG
VRTN	1	TGACGCTGTAGCGCTCACAG
	2	AAAGCACCTTCTACCGCTGG
	3	GCACCTTCTACCGCTGGCGG
	4	CCAAAAGCACCTTCTACCGC
WDR33	1	CTAGTGGGGAGTTTACCCTG
	2	CTTGGCATATGGAAAAAACG
	3	GGCATATGGAAAAAACGAGG
	4	AAAGAGACCAGAGAGATATG
ZFR	1	AAGTTGGAACTCGCTGACAG
	2	AATGCGTATGATAATCACAC
	3	GCAAAAGGATTACTTCTCCG
	4	AATAATTTTGAATTCATGTG
ZIC2	1	GGACCGTGAACTGAGCCTGG
	2	CTCGGAGCAATACCGCCAGG
	3	CGCAGCCCTCAAACTCACAC
	4	GCTTCGCCAACAGCAGCGAC
ZNF593	1	TTCGACCCCGACCTGCCAGG
	2	CCCGGCAGATGAAGGCGAAG
	3	TCGGAAGTGGGTCTTCAGGT
	4	GAAGTGGGTCTTCAGGTTGG
NT	1	ACGGAGGCTAAGCGTCGCAA
	2	CGCTTCCGCGGCCCGTTCAA
	3	ATCGTTTCCGCTTAACGGCG
	4	GTAGGCGCGCCGCTCTCTAC
	5	CCATATCGGGGCGAGACATG
	6	TACTAACGCCGCTCCTACAG
	7	TGAGGATCATGTCGAGCGCC
	8	GGGCCCGCATAGGATATCGC

	9	TAGACAACCGCGGAGAATGC
	10	ACGGGCGGCTATCGCTGACT
	11	CGCGGAAATTTTACCGACGA
	12	CTTACAATCGTCGGTCCAAT
	13	GCGTGCGTCCCGGGTTACCC
	14	CGGAGTAACAAGCGGACGGA
	15	CGAGTGTTATACGCACCGTT
	16	CGACTAACCGGAAACTTTTT

Table S3. Sequence of qRT-PCR Primers.

Target Gene	Forward Primer	Reverse Primer
CARHSP1	CCCGTCTACAAAGGAGTCTGCA	ATTCTTGGGTGGGATGGAGCAC
ASF1A	GGTGCTGGATAACCCTTCTCCTT	GAACAGGACCCACTAAAACAGAGTC
ZIC2	GCGCAACTCCACAACCAGTA	CGATCCACTTGCAGATTAGCTCC
SALL4	CCAGCACATCAACTCGGAGGA	CATTCCCTGGGTGGTTCACTG
ZNF8	TCCCGACAGATGCTCCTTATCC	GAGGCCAAATTCCAACTGCTTCA
UVRAG	GCCAGACCGTCTTGATACATCTG	CTGACCCAAGTATTTCAGCCCA
OCT4	CGACCATCTGCCGCTTTGAG	GCACGAGGGTTTCTGCTTTGC
SOX2	CTACAGCATGTCCTACTCGCAGC	GGAGGAAGAGGTAACCACAGGG
NANOG	CCCCAGCCTTTACTCTTCCTACC	AGGTCTGGTTGCTCCACATTGG
KLF2	CCTACACCAAGAGTTCGCATCTG	TCTGAGCGCGCAAACTTCCAG
KLF4	CGGACATCAACGACGTGAGC	CCTTCAGCACGAACTTGCCC
KLF17	GGGGCCACAGTTCAGTATGC	CTCTTGCTGAGGGGGAGACATTC
TFCP2L1	CACTCAGCCAGCTGCCAGAT	GGTGGTTTCATAGGACGGCTGG
DPPA3	CAACCTACATCCCAGGGTCTCC	CTCGGAGGAGATTTGAGAGGCC
DNMT3L	CTGCGGAAGTCTCCAGGTTCAC	CGTCATCGTCGTACAGGAAGAGG

Table S4. Information of Antibodies.

Materials	Source	Identifier
Goat polyclonal anti-FOXA2	R&D Systems	#AF2400
Anti-human NANOG	R & D	#AF1997
Anti-OCT-3/4 Antibody	Santa Cruz	#sc-5279
Anti-SOX2 (D6D9)	Cell Signaling	#3579
Donkey anti-Mouse IgG (H+L) Highly Cross-Adsorbed Secondary Antibody, Alexa Fluor 488	Thermo Fisher Scientific	#A-21202
Donkey anti-Rabbit IgG (H+L) Secondary Antibody, Alexa Fluor 594 conjugate	Thermo Fisher Scientific	#A-21207
Donkey anti-Goat IgG (H+L) Cross-Adsorbed Secondary Antibody, Alexa Fluor 647	Thermo Fisher Scientific	#A-21447
Donkey anti-Goat IgG Secondary Antibody, Alexa Fluor 594	Thermo Fisher Scientific	#A-32816
Donkey anti-Rabbit IgG Secondary Antibody, Alexa Fluor 647	Thermo Fisher Scientific	#A-32795
